# The crosstalk of ABCA1 and ANXA1: a potential mechanism for protection against atherosclerosis

**DOI:** 10.1186/s10020-020-00213-y

**Published:** 2020-09-07

**Authors:** Xin Shen, Shun Zhang, Zhu Guo, Dongming Xing, Wujun Chen

**Affiliations:** 1 Cancer Institute, The Affiliated Hospital of Qingdao University, Qingdao University, Qingdao Cancer Institute, Qingdao, 266071 Shandong China; 2 Department of Spine Surgery, The Affiliated Hospital of Qingdao University, Qingdao, 266071 Shandong China; 3 School of Life Sciences, Tsinghua University, Beijing, 100084 China

**Keywords:** ANXA1, ABCA1, PPARγ, Crosstalk, Atherosclerosis

## Abstract

Atherosclerosis, characterized by the formation of fat-laden plaques, is a chronic inflammatory disease. ABCA1 promotes cholesterol efflux, reduces cellular cholesterol accumulation, and regulates anti-inflammatory activities in an apoA-I- or ANXA1-dependent manner. The latter activity occurs by mediating the efflux of ANXA1, which plays a critical role in anti-inflammatory effects, cholesterol transport, exosome and microparticle secretion, and apoptotic cell clearance. ApoA-I increases ANXA1 expression via the ERK, p38MAPK, AKT, and PKC pathways. ApoA-I regulates the signaling pathways by binding to ABCA1, suggesting that apoA-I increases ANXA1 expression by binding to ABCA1. Furthermore, ANXA1 may increase ABCA1 expression. ANXA1 increases PPARγ expression by modulating STAT6 phosphorylation. PPARγ also increases ANXA1 expression by binding to the promoter of ANXA1. Therefore, ABCA1, PPARγ, and ANXA1 may form a feedback loop and regulate each other. Interestingly, the ANXA1 needs to be externalized to the cell membrane or secreted into the extracellular fluids to exert its anti-inflammatory properties. ABCA1 transports ANXA1 from the cytoplasm to the cell membrane by regulating lipidization and serine phosphorylation, thereby mediating ANXA1 efflux, likely by promoting microparticle and exosome release. The direct role of ABCA1 expression and ANXA1 release in atherosclerosis has been unclear. In this review, we focus on the role of ANXA1 in atheroprogression and its novel interaction with ABCA1, which may be useful for providing basic knowledge for the development of novel therapeutic targets for atherosclerosis and cardiovascular disease.

## Introduction

Cardiovascular diseases (CVDs), including coronary artery disease (CAD), myocardial infarction (MI), and heart failure (HF), are the leading cause of death in humans and are manly caused by atherosclerosis. Atherosclerosis, characterized by the formation of fat-laden plaques in large- and medium-sized vessels, has been identified as a chronic inflammatory disease of the artery wall (Saigusa et al. 2020). Atherosclerosis occurs through the recruitment of monocytes and their differentiation into macrophages, which require lipids for foam cell generation, a process regulated by balancing the rates of lipid uptake and efflux (Peng et al. 2020). The latter is mainly controlled by ATP binding cassette transporter A1 (ABCA1), a membrane transporter that is abundant in macrophages and mediates cholesterol efflux (Wang et al. 2020). ABCA1 promotes the efflux of free cholesterol to apolipoprotein A-I (apoA-I) and is critical for high-density lipoprotein (HDL) particle biogenesis. These particles protect against atherosclerotic vascular diseases by transferring cholesterol from peripheral cells to the liver for biliary excretion, constituting the process of reverse cholesterol transport (Libby et al. 2019; Ahmadi et al. 2019).

Recently, several studies have shown that ABCA1 reduces inflammatory responses by inhibiting the secretion of interleukin-1β (IL-1β) and tumor necrosis factor (TNF-α) and the NF-κB signaling pathway via the removal of reactive oxygen species (ROS) (Babashamsi et al. 2019), and ABCA1 may be linked to these functions via apoA-I. Interestingly, ABCA1, in addition to mediating cholesterol efflux, promotes the secretion of *annexin A1* (*ANXA1*), which is associated with anti-inflammatory reactions, cell differentiation and proliferation, cell phagocytosis and clearance, cholesterol homeostasis, immunogenic cell death (ICD), autophagy, apoptosis, and exosomes (Chapman et al. 2003; Omer et al. 2006; Li et al. 2018). ANXA1 is also a specific marker of microparticles, and accumulating evidence has shown that ANXA1 plays a key role in inhibiting the development of atherosclerosis via an anti-inflammatory reaction (Jeppesen et al. 2019). The ANXA1 mimetics Ac2–26 and CGEN-855A have also been shown to protect against atherosclerosis and MI progression via an anti-inflammatory reaction. CGEN-855A (TIPMFVPESTSKLQKFTSWFM-amide) is a novel 21-amino acid mimetic peptide of ANXA1. CGEN-855A was developed by Compugen Ltd., Tel Aviv, Israel (Hecht et al. 2009; Qin et al. 2015). The anti-inflammatory and cardio protective effects of CGEN-855A have been investigated by pharmaceutical companies, but the direct effects of ANXA1 on cholesterol transport, exosome and microparticle secretion, apoptotic cell clearance, and ICD in atherosclerosis have rarely been investigated. Although ABCA1 mediates ANXA1 release, the effect of this function on atherosclerosis has been unclear. In addition, ANXA1 may increase ABCA1 expression, but the effect on atherosclerosis development is unknown (Zhou et al. 2009; da Rocha et al. 2019; Chawla et al. 2001; Parente and Solito 2004). In this article, we review the roles of ANXA1 in atherosclerosis and focus on the crosstalk of ABCA1 and ANXA1. We expect to promote the study of the role of ANXA1 in atherosclerosis and sincerely hope that more scientists will study the relationship of ABCA1 with ANXA1 and atherosclerosis development.

## ANXA1 mediates anti-inflammatory effects, cholesterol transport, exosome and microparticle secretion, and apoptotic cell clearance

ANXA1, the first identified member of the annexin family, is an anti-phospholipase protein that has Ca^2+^ and phospholipid binding sites and high biological and structural homology to other family members (Parente and Solito 2004). ANXA1 is highly expressed in neutrophils and monocytes/macrophages, and its anti-inflammatory activity is associated with interference of the leukocyte migration and platelet aggregation (Parente and Solito 2004; Senchenkova et al. 2019). ANXA1 was predominantly found within gelatinase granules and in the cytoplasm (Perretti et al. 2000; Solito et al. 2006). ANXA1 binds to and activates the formyl peptide receptor (FPR) on leukocytes and endothelial cells, thereby inhibiting leukocyte migration to inflammatory sites (Senchenkova et al. 2019; Parisi et al. 2019; Pessolano et al. 2019). ANXA1 also reduces inflammatory responses by inhibiting phospholipase A_2_ (PLA_2_), thereby inhibiting the synthesis of arachidonic acid (AA) and AA-derived metabolites, including thromboxane A2 (TXA_2_), prostaglandin E_2_ (PGE_2_), PGI_2_, PGD_2_, and PGF2α (Sanches et al. 2020; Seidel et al. 2012). ANXA1 also prevents inflammatory responses by reducing nitric oxide synthase (iNOS) and the expression of cyclooxygenase-2 (COX-2), which is a rate-limiting key enzyme of prostaglandin synthesis (Seidel et al. 2012; Kiani-Esfahani et al. 2016). ANXA1 also induces macrophage polarization toward a more anti-inflammatory phenotype that secretes IL-10, an anti-inflammatory cytokine (Ferlazzo et al. 2003; Butcher and Galkina 2015; Giannarelli and Wong 2019). Thus, ANXA1 functions as an anti-inflammatory protein via multiple mechanisms, and many studies suggest that ANXA1 reduces atherosclerosis progression via an anti-inflammatory reaction. In human coronary atherosclerotic plaques, ANXA1 was found to localize in macrophages and endothelial cells in the tunica intima (de Jong et al. 2017a). The expression of the ANXA1 gene in the carotid plaques of asymptomatic patients was significantly higher than in symptomatic patients (Cheuk and Cheng 2011). The actions of ANXA1, which are mediated through its receptor FPR2, can be mimicked by an amino-terminal peptide encompassing Ac2–26, demonstrating a protective effect of ANXA1 on the early stage of plaque development (Kusters et al. 2015; de Jong et al. 2017b).

Recently, apoA-I was found to increase ANXA1 expression via the ERK, p38 MAPK, AKT, and PKC pathways in human umbilical vein endothelial cells (HUVECs) (Pan et al. 2016). ApoA-I can bind to ABCA1 and then influence several signaling pathways, including ERK, p38 MAPK, AKT, and PKC, and the synthesis of apoA-I protein is generally thought to occur in the liver and small intestine. However, apoA-I is also transcribed in macrophages, and the endogenous apoA-I in macrophages has an anti-inflammatory effect, suggesting that ABCA1 increases ANXA1 expression through binding to apoA-I, controlling the ERK, p38 MAPK, AKT, and PKC pathways (Chen et al. 2020). The drugs associated with apoA-I that are used to treat CVD, particularly CAD, have an apoA-I mimetic peptide, including D-4F, L-4F, 6-F, 18A, 37pA, 5A ETC-642, FAMP, apoA-I milano including MDCO-216 (Phase 2, Termination), rHDL particles that comprise human plasma-derived apoA-I and soybean phosphatidylcholine including CSL111 (Phase 2) and CSL112 (Phase 3), HDL mimetics that comprise recombinant full-length human apoA-I and two natural phospholipids including CER-001 (Phase 2), and BET-inhibitor including RVX-208, which significantly stimulate apoA-I transcription (Phase 3) (Yu et al. 2019). Many studies have shown that these drugs have an anti-inflammatory effect by increasing apoA-I expression (Yu et al. 2019). Based on the observed strong activation of apoA-I with ANXA1 expression, these drugs may augment the anti-inflammatory effect by increasing the apoA-I/ANXA1 axis. Therefore, we hypothesize that the drugs associated with apoA-I have multiple functions stimulating the apoA-I/ANXA1 axis, including anti-inflammatory effects, cholesterol transport, exosome and microparticle secretion, apoptotic cell clearance, and ICD. According to this hypothesis, new drug targets may exist, but more research is needed.

Many studies have shown a relationship between ANXA1 and lipid metabolism. ANXA1 expression is strongly increased in adipose tissue, including in mice fed a high-fat diet (Akasheh et al. 2013) and obese children (Aguilera et al. 2015), and in the subcutaneous fat of young and old overweight patients (Alfadda et al. 2013). ANXA1 expression is also increased in the livers of mice with nonalcoholic steatohepatitis (NASH), which is characterized by hepatic lipid accumulation (Locatelli et al. 2014). The plasma levels of ANXA1 in patients with type1 diabetes (T1D) and T2D are significantly higher than in control patients. In addition, the levels of ANXA1 in T2D patients are positively correlated with serum low-density lipoprotein cholesterol (LDL-C) levels, total cholesterol levels, and the fatty liver index (FLI). ANXA1−/− mice have significantly higher serum triglyceride levels, more extensive Oil Red-O staining in the liver, and are more obese than wild-type mice, and administration of hrANXA1 to these mice reduces serum cholesterol and liver triglyceride levels, the extent of Oil Red-O staining in liver, and weight gain (Purvis et al. 2019a). These findings may suggest that ANXA1 is associated with lipid and glucose metabolism. Moreover, ANXA1 mediates cholesterol transport from the endoplasmic reticulum (ER) to multivesicular bodies (MVB) and then stimulates the secretion of exosomes from MVBs via endosomal-sorting complexes required for transport (ESCRT) and Alg-2 interacting protein X (Alix) (Eden et al. 2016; Rentero et al. 2018). Interestingly, exosomes also carry ANXA1 (Rentero et al. 2018; Raulf et al. 2018). Exosomes protect and transport lipids, proteins, and RNAs, fostering intercellular communication among different cell types involved in atherosclerosis, such as macrophages, endothelial cells, and smooth muscle cells. Many studies suggest that exosomes could be used for not only the diagnosis but also the treatment of atherosclerosis (Reiss et al. 2017; Wang et al. 2019), but whether the interaction of ANXA1 with exosomes also regulates atherosclerosis progression needs to be further studied.

ANXA1 was also found in microparticles (also known as microvesicles) from complex vesicular structures shed from endothelial cells, insulin-secreting Rin-m5f β-cells, ATRA-NB4 cells, neutrophils, and leukocytes (Kreutter et al. 2017; Nadkarni et al. 2011; Tsai et al. 2012; Tsai et al. 2013), and it serves as a specific marker of microparticles shed from the plasma membrane (Jeppesen et al. 2019). However, the role of ANXA1 in microparticles has not been investigated. Microparticles, heterogeneous extracellular vesicles that include exosomes, nanoparticles, and shedding vesicles, are not only a prognostic marker of atherosclerosis acceleration and clinical presentation of familial hypercholesterolemia but also therapeutic agents for CAD (Boulanger et al. 2017; Loyer et al. 2014; Suades et al. 2019). Therefore, ANXA1 could regulate atherosclerosis development by mediating the transport of cholesterol and the secretion of exosomes and microparticles. However, the relationship between ANXA1-mediated cholesterol transport and the secretion of exosomes and microparticles is unclear.

ANXA1 is also an ICD hallmark. The ANXA1 secreted from apoptotic cells binds to the FRP1 receptor on antigen-presenting cells (APCs) and then helps guide the APCs to the dying cells to promote their clearance (Cruickshank et al. 2018). Many studies have demonstrated that impaired clearance of apoptotic cells promotes the development of atherosclerosis, suggesting that the ICD process by ANXA1 may play a key role in atherosclerosis development (Aprahamian et al. 2004; Poon et al. 2014). Intensive studies of ICD have focused on cancer, leaving its role in atherosclerosis development largely unelucidated.

## The crosstalk between ABCA1 expression and ANXA1 efflux

ANXA1 secretion has been found to occur in many types of cells, including monocytes/macrophages, mast cells, epithelial cells, folliculo-stellate (FS) cells, neutrophils, and astrocytes (Purvis et al. 2019b; Sugimoto et al. 2016). It has also been detected in a variety of tissues, such as heart, artery, skeletal muscle, small intestine, colon, adipose, kidney, liver, lung, spleen, stomach, pancreas, skin, brain, and prostate tissue, wherein ABCA1 is strongly expressed (Frambach et al. 2020). In addition, the expression of ABCA1 and ANXA1 are positively correlated in plaque macrophages with oxidized low-density lipoprotein (oxLDL) and monocytes of hypercholesterolemic pigs (Geeraert et al. 2007; Orekhov et al. 2018). Traditionally, ABCA1 has been mainly found to promote cholesterol efflux, and its anti-inflammatory effect is associated with apoA-I (Phillips 2018). Previous studies from our laboratory and others have demonstrated that the ABCA1 complex with apoA-I plays a critical role in cholesterol efflux and anti-inflammatory activities (Kuang et al. 2017; Yin et al. 2012; Chen et al. 2013; Tang et al. 2012; Price et al. 2019). Interestingly, ANXA1 secretion has been shown to require ABCA1 in macrophages and endocrine cells. The ABCA1 inhibitor glyburide blocks ANXA1 secretion (Seidel et al. 2012). Chapman et al. revealed that ABCA1 promotes ANXA1 release in mouse FS and A549 cells. ANXA1 interacts with ABCA1 via ANXA1 amino acids 196 to 274 and colocalizes with ABCA1 in the cell plasma membrane (Chapman et al. 2003), and an ABCA1 inhibitor and siRNA were shown to significantly reduce the membrane localization of ANXA1. In contrast, ABCA1 overexpression in AtT20 D1 FS cells significantly increases plasma membrane-associated ANXA1 expression (Omer et al. 2006). Importantly, ABCA1 mediated the secretion of ANXA1 in Hensen cells, in which the majority of ANXA1 is stored inside lipid droplets. Myosin II promotes ANXA1 translation from lipid droplets to the apical region of Hensen cells, in which ABCA1 facilitates the release of ANXA1 to the external milieu (Kalinec et al. 2009). Of note, ischemia-reperfusion injury increases ABCA1 ubiquitination and degradation in the ganglion cell layer, leading to decreased ANXA1 translocation to the cell membrane, and its secretion facilitates retinal inflammation and retinal ganglion cell apoptosis (Xiao et al. 2018). Notably, the LXR agonist T0901317 induces ICD by increasing calreticulin (CRT) and high mobility group protein (HMGB1) in colon cancer cells (Wang et al. 2018a). ABCA1 is a target gene of LXR. T0901317 also increases ABCA1 expression. ABCA1 promotes ANXA1 release, suggesting that the ABCA1/ANXA1 axis plays a critical role in the ICD by T0901317. Taken together, these data show that ABCA1 promotes the efflux of ANXA1, which is critical for the activities of ANXA1.

Importantly, the ANXA1 needs to be externalized to the cell membrane or secreted into the extracellular fluids to exert its anti-inflammatory properties. The transport of ANXA1 from the cytoplasm to the cell membrane is dependent on serine phosphorylation and lipidization (Solito et al. 2006). ABCA1 is a key regulator of apoA-I (Wang et al. 2013) and apoE lipidation (Krimbou et al. 2004), and ABCA1 also contains serine residues in its intracellular segment; the phosphorylation of ABCA1 serine residues can increase cholesterol efflux (Hu et al. 2009). ABCA1 mediates the secretion of ANXA1, which is stored in lipid droplets and localized in microparticles and exosomes. ABCA1 also mediates microparticle (Duong et al. 2006; Nandi et al. 2009; Hafiane and Genest 2017) and exosome secretion (Hafiane and Genest 2017; Ma et al. 2011). ABCA1 transports ANXA1 from the cytoplasm to the cell membrane, likely by regulating lipidization and serine phosphorylation, thereby mediating ANXA1 efflux most likely by promoting microparticle and exosome release. However, the role of the colocalization of both proteins on the plasma membranes during ANXA1 efflux needs to be determined. This hypothesis may be correct, at least ABCA1 transports ANXA1 from the cytoplasm to the cell membrane. Thus, ABCA1-mediated ANXA1 transport and release play a key role in the anti-inflammatory action of ANXA1.

Interestingly, enhanced AA production through upregulation of cPLA_2_ activity has been shown to inhibit LXR expression and thereby reduce ABCA1 expression and cholesterol efflux (Zhou et al. 2009). As mentioned before, ANXA1, which serves as the inhibitory protein of PLA2, can reduce the synthesis of AA. ANXA1−/− mice have shown increased serum triglyceride levels and Oil Red-O staining of the liver as well as enhanced obesity compared with wild-type mice. Administration of hrANXA1 to mice reduced serum cholesterol and liver triglyceride levels, Oil Red-O staining in the liver, and weight gain, suggesting that ANXA1 increases ABCA1 expression and cholesterol efflux. In addition, peroxisome proliferator-activated receptor-gamma (PPARγ), which has been implicated in lipid metabolism, the inflammatory response, and glucose homeostasis, is increased in ANXA1−/− mice, suggesting a role for ANXA1 in mediating PPARγ expression (Akasheh et al. 2013). ANXA1 increases PPARγ and CD36 expression by modulating STAT6 phosphorylation in BV2 cells, subsequently mediating phagocytosis of apoptotic cells (da Rocha et al. 2019). Macrophage CD36 participates in atherosclerotic arterial lesion formation via the promotion of oxLDL uptake and foam cell formation. However, ABCA1 and CD36 are induced by PPARγ activation. Thus, PPARγ controls intracellular cholesterol balance, including cholesterol uptake, processing, and cholesterol removal, through ABCA1 and CD36 (Chawla et al. 2001). IL-10 also increases CD36 and ABCA1 expression, thereby facilitating cholesterol uptake (minor) and efflux (major) (Han et al. 2009). IL-19, which has a similar exon-intron structure and shares a 21% amino acid identity with IL-10, also increases ABCA1 and ABCG1 expression and cholesterol efflux (Gabunia et al. 2016). Administration of 10 ng/g/day IL-19 to LDLR−/− mice almost completely inhibits plaque formation in the aortic arch, and IL-19 at as little as 1 ng/g/day reduces the plaque area by 70%, suggesting that IL-19 has a strong anti-atherosclerotic effect (Ellison et al. 2013). Mechanistic studies have shown that IL-19 reduces cytokine-induced inflammation by promoting the activation of STAT3, STAT6, Kruppel-like factor 4 (KLF4), and PPARγ (Gabunia et al. 2016). IL-19 regulates lipid metabolism via the PPARγ-dependent regulation of CD36-mediated cholesterol uptake and ABCA1-mediated cholesterol efflux (Gabunia et al. 2016). IL-19 reduces oxLDL uptake by inducing the expression of miR-133a to reduce the expression of the target gene low-density lipoprotein receptor adaptor protein 1 (LDLRAP1), which functions to internalize the LDL receptor (Ellison et al. 2013; Gabunia et al. 2017). IL-19 reduces TNFα expression by inducing the expression of miR-133a to reduce the expression of human antigen R (HuR), which is an important regulator of TNFα mRNA stability (Ray et al. 2018), suggesting that IL-19 is a link between inflammation and cholesterol metabolism. In addition, ANXA1 decreases the expression levels of some miRNAs in MCF-7 cells, including miR-26b*, miR-27a, miR-200c, and miR-562 (Anbalagan et al. 2014). Previous studies by our laboratory and others have demonstrated that miRNAs, including miR-27a and miR-10b, decrease cholesterol efflux by binding to the 3’UTR of ABCA1 (Zhang et al. 2014; Wang et al. 2018b; Wu et al. 2020). Based on the aforementioned information, we hypothesize that ANXA1 can also increase ABCA1 expression by regulating the expression of PPARγ, IL-10, and miRNAs, and then regulate cholesterol uptake and efflux. Given that ANXA1 is a protective factor for atherosclerosis, its role in cholesterol efflux may be dominant. Interestingly, PPARγ has also been shown to increase ANXA1 expression by binding to the promoter of ANXA1 in breast cancer cells (Chen et al. 2017). Thus, ABCA1, PPARγ, and ANXA1 may form a feedback loop and regulate each other. Notably, PPARγ induces cholesterol efflux by binding to LXRa and controlling the expression of the target gene ABCA1 (Wang et al. 2018c; Tsuboi et al. 2020), suggesting that PPARγ may play a primary role in the feedback loop. However, this interaction among ABCA1, PPARγ, and ANXA1 requires further study.

## Summary

**Fig. 1:**
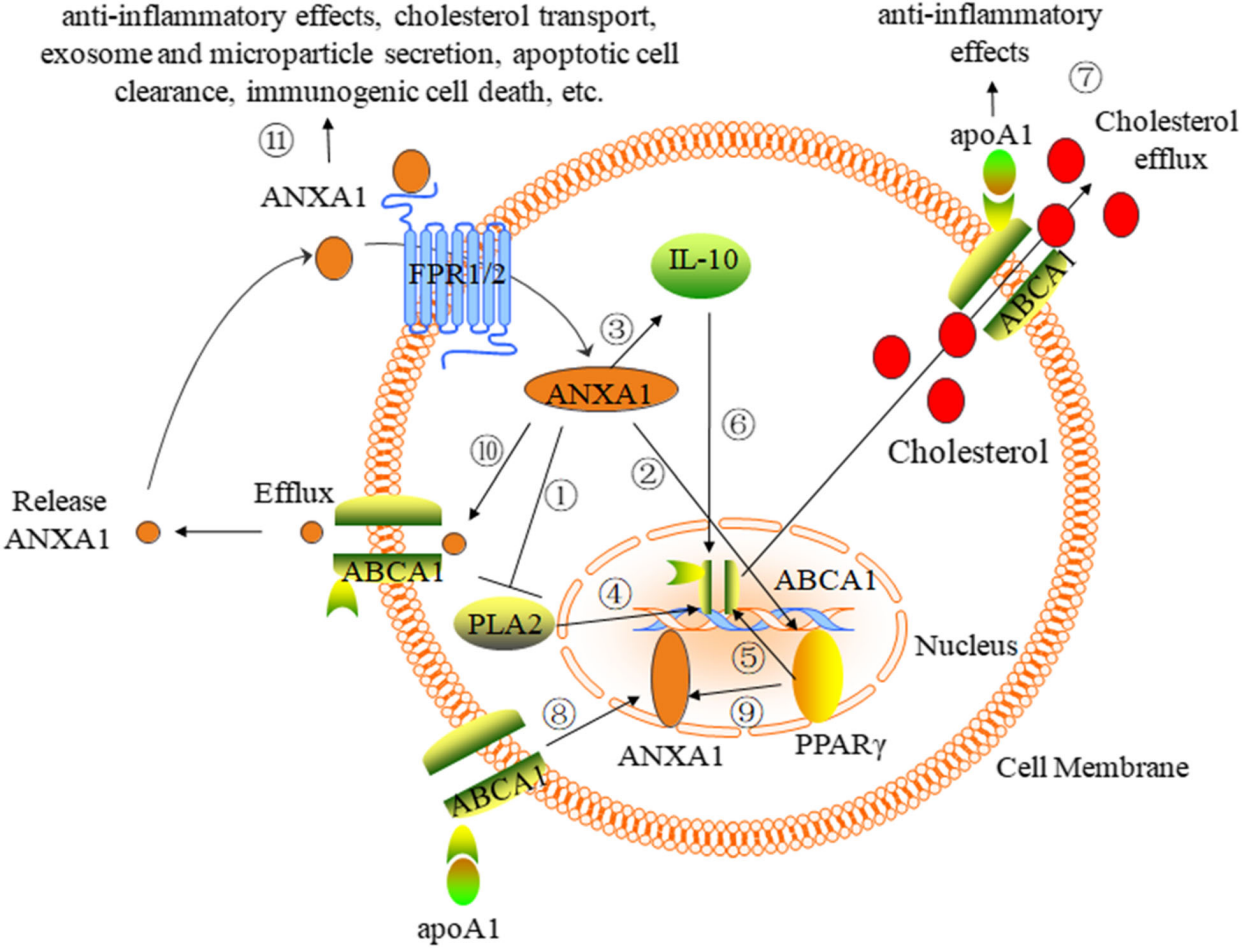
The mechanism and function of the ABCA1 interaction with ANXA1. ANXA1 serves as an inhibitory protein of PLA2 (①) and increases the expression of PPARγ (②), IL-10 (③). The expression of ABCA1 is increased by the PLA_2_ inhibitory protein (④), PPARγ (⑤), IL-10 (⑥). Therefore, ANXA1 increases ABCA1 expression and then combines with apoA-I to promote cholesterol efflux and anti-inflammatory effects (⑦). However, this function has not been investigated and requires further study. ABCA1 increases ANXA1 expression by binding to apoA-I, controlling the ERK, p38 MAPK, AKT, and PKC pathways (⑧), and PPARγ increases ANXA1 expression by binding to the promoter of ANXA1 (⑨). ABCA1 transports ANXA1 and thereby mediates ANXA1 efflux and release (⑩), thereby promoting the functions of ANXA1, including anti-inflammatory effects, cholesterol transport, exosomes, microparticle secretion, apoptotic cell clearance, and immunogenic cell death (⑪). Thus, ABCA1, PPARγ, and ANXA1 may form a feedback loop and regulate each other. However, this interaction among ABCA1, PPARγ, and ANXA1 requires further study

ANXA1-mediated anti-inflammatory effects, cholesterol transport, exosome and microparticle secretion, and apoptotic cell clearance play key roles in atheroprogression. Importantly, ABCA1, in addition to mediating cholesterol efflux and anti-inflammatory effects by binding to apoA-I, increases ANXA1 expression and transports ANXA1 from the cytoplasm to the cell membrane, thereby promoting ANXA1 into the extracellular fluids to facilitate anti-inflammatory activities and apoptotic cell clearance. ANXA1 may also increase ABCA1 expression and cholesterol efflux by serving as the PLA_2_ inhibitory protein or increasing PPARγ and IL-10 expression. ABCA1, PPARγ, and ANXA1 may form a feedback loop and regulate each other (Fig. 1). Despite this evidence for the relationship of mutual regulation between ABCA1 expression and ANXA1 efflux, less is known regarding the potential role of this mutual regulatory effect in cardiovascular disease. Further studies need to focus on the role of ABCA1-mediated ANXA1 efflux and ANXA1-mediated ABCA1 expression in the development of atherosclerosis. In addition, ABCA1 is not the only transporter that mediates ANXA1 secretion, as ABCC1 also mediates this phenomenon (Wein et al. 2004). The relationship between ABCA1 and ABCC1 during ANXA1 efflux requires further study. Taken together, these observations suggest that crosstalk may exist between ANXA1 and ABCA1, IL-10, and PPARγ. However, the direct association of ANXA1 with increased ABCA1 expression has not been investigated. Finally, we sincerely hope that many more scientists will focus on crosstalk in atherosclerosis.

## Data Availability

Not applicable.

## Abbreviations

CVD: Cardiovascular disease
CAD: Coronary artery disease
MI: Myocardial infarction
HF: Heart failure
ABCA1: ATP binding cassette transporter A1
ApoA-I: Apolipoprotein A-I
HDL: High-density lipoprotein
IL-1: Interleukin-1
TNF-α: Tumor necrosis factor
ROS: Reactive oxygen species
*ANXA1*: *Annexin A1*
ICD: Immunogenic cell death
FPR: Formyl peptide receptor
PLA_2_: Phospholipase A_2_
AA: Arachidonic acid
TXA_2_: Thromboxane A_2_
PGE_2_: Prostaglandin E_2_
iNOS: Nitric oxide synthase
COX-2: Cyclooxygenase-2
ER: Endoplasmic reticulum
MVB: Multivesicular bodies
ESCRT: Endosomal-sorting complexes required for transport
APCs: Antigen-presenting cells
oxLDL: Oxidized low-density lipoprotein
FS: Folliculo-stellate
PPARγ: Peroxisome proliferator-activated receptor-gamma
